# A Robotic Solution for Precision Smoothing and Roughening of Precast Concrete Surfaces: Design and Experimental Validation

**DOI:** 10.3390/s24113336

**Published:** 2024-05-23

**Authors:** Rui Gang, Zhongxing Duan, Lin Wang, Lemeng Nan, Jintao Song

**Affiliations:** 1 College of Information and Control Engineering, Xi’an University of Architecture and Technology, Xi’an 710311, China; grui@xauat.edu.cn (R.G.); nanlm@xauat.edu.cn (L.N.); 15738941226@xauat.edu.cn (J.S.); 2 School of Mechanical and Electrical Engineering, Xi’an University of Architecture and Technology, Xi’an 710311, China; linwang@xauat.edu.cn

**Keywords:** prefabricated construction, the smoothing and roughening robot, multi-degree-of-freedom integrated intelligent end-effector, machining path planning methods

## Abstract

Prefabricated construction has pioneered a new model in the construction industry, where prefabricated component modules are produced in factories and assembled on-site by construction workers, resulting in a highly efficient and convenient production process. Within the construction industry value chain, the smoothing and roughening of precast concrete components are critical processes. Currently, these tasks are predominantly performed manually, often failing to achieve the desired level of precision. This paper designs and develops a robotic system for smoothing and roughening precast concrete surfaces, along with a multi-degree-of-freedom integrated intelligent end-effector for smoothing and roughening. Point-to-point path planning methods are employed to achieve comprehensive path planning for both smoothing and roughening, enhancing the diversity of textural patterns using B-spline curves. In the presence of embedded obstacles, a biologically inspired neural network method is introduced for precise smoothing operation planning, and the A* algorithm is incorporated to enable the robot’s escape from dead zones. Experimental validation further confirms the feasibility of the entire system and the accuracy of the machining path planning methods. The experimental results demonstrate that the proposed system meets the precision requirements for smoothing and offers diversity in roughening, affirming its practicality in the precast concrete process and expanding the automation level and application scenarios of robots in the field of prefabricated construction.

## 1. Introduction

Prefabricated construction has long been widely recognized as an effective alternative to traditional building methods [1,2,3]. It offers significant potential advantages, including but not limited to enhanced material utilization [4], safety, labor productivity, craftsmanship, workflow continuity, and reduction in time, cost, and waste [5]. However, the broader adoption of prefabricated construction faces various barriers, including reliance on conventional methods [6], underutilization of factory space, complexity in workflow processes, fragmentation of information, cost barriers [7], and inconsistency in quality [5].

Prefabricated construction primarily follows an industrial production model characterized by “standardized design—factory manufacturing—onsite assembly” [8]. It is categorized into three major systems based on the main structure material: wooden structure, steel structure, and PC (Precast Concrete) component structure [9]. Steel structure manufacturing primarily entails cutting and welding, while wooden component manufacturing mainly involves mechanical processing, both of which exhibit a high degree of production automation. However, due to the composite material characteristics of steel-reinforced concrete, the automation level of PC components is not yet fully developed. This paper specifically focuses on the manufacturing of PC components [10].

The production processes for PC components primarily include the transportation of concrete, formwork setting, concrete pouring, compaction and molding, smoothing/roughening, concrete curing, formwork dismantling, and inspection. Smoothing and roughening are processes that occur after concrete is poured and leveled [11]. During the initial setting stage of the concrete, the surface is compacted to expel excess water, then scraped again to make it smooth [12]. Traditionally, workers have to squat on the ground and use a steel trowel to make the concrete surface flat, smooth, and mark-free, which is time-consuming and labor-intensive, and the consistency of operation and the smoothness of the concrete surface are difficult to ensure due to the varying skill levels of workers [13]. If robots are used for concrete surface operations, the workload will be greatly reduced, and the surface quality will be more uniform. In recent years, the application of robots and related technologies on PC component production lines has become increasingly common. Based on the application scenarios, the existing precast concrete robots can be classified into concrete distribution robots [14,15], concrete compaction robots [16,17,18], concrete smoothing robots [19], concrete surface coating robots [20], and construction monitoring robots [21,22].

Woo et al. [23] designed a small-scale concrete finishing robot, which consists of a central unit with an electrically-driven mobile platform. These units are located at the front and back of the central unit, while the measurement and control units are positioned at the top of the mobile platform’s central unit. The end effector is mounted at the bottom of the working unit and is responsible for surface finishing tasks. This end effector utilizes an ultrasonic vibration unit connected to a trowel to achieve smoothing effects by pressing the concrete surface. However, the operation of this robot primarily relies on remote control by workers rather than operation through pre-programmed motion paths. Therefore, the workers’ operational skills significantly affect the smoothness of the concrete surface.

Currently, only a few manufacturers offer solutions for concrete surface finishing in the market. For example, the smoothing machine developed by Allen Engineering Corporation is driven by workers for the concrete finishing process [24]. Beneath the machine, there are two sets of high-speed, high-torque rotating mud pans. Each blade forms a slight angle with the ground and, under high-speed rotation, presses tightly against the concrete surface, squeezing out water and filling surface gaps. Additionally, it is equipped with standard features such as power steering control and jacks to ensure that workers can easily maneuver and turn indoors. Operators of the driving machine need skills to operate it and must endure the intense vibrations from the engine and trowel. The large-span smoothing machine for multi-mold operations was introduced by Avermann in Germany [25] and the intelligent smoothing machine for multi-end effector collaborative operations was developed by Vollert [26]. Both manufacturers’ smoothing devices include leveling and compacting mechanisms. The leveling mechanism, controlled by a motor, is designed to disperse excess concrete material accumulated in the front, forming a primary mortar surface. The motor drives a ball screw to rotate, further pushing the compacting mechanism’s plate to vertically strike the concrete surface. This device only requires remote operation by workers, reducing labor intensity. However, the precision of smoothing is influenced by the operator’s experience, potentially leading to variations in accuracy. Typically, the smoothing accuracy per meter falls within an error range of 10 to 15 mm. Bright Dream Robotics, based in Guangdong, China, has developed the basement smoothing robot [27]. It adopts the blade-driven mode of traditional manual smoothing machines to achieve intelligent operation and integrates navigation technology and path planning algorithms. The robot has two operating modes: remote control and automatic. In automatic mode, the robot operates at the four corners of the work surface to achieve coverage. When encountering protruding steel bars or pipes, the robot cannot move and requires manual assistance. After using this robot for smoothing operations, the surface smoothness of the concrete typically ranges around 9 mm per meter, with an efficiency improvement of approximately 30% compared to traditional manual methods.

After researching solutions from several construction robot manufacturers, it is found that the smoothing robots available in the market can replace manual operations to achieve precise smoothing, thereby improving construction efficiency. However, there are still some issues: the control algorithms are too simplistic, allowing only for fixed operation routes, making it incapable of covering large areas, and it is unable to handle components with embedded steel bars and conduits, which impact subsequent construction processes. For components without embedded items, automation ensures consistent surface integrity. However, components with embedded items present challenges for traditional robots in achieving precise control and repeatability, which are crucial for avoiding obstacles and ensuring complete surface coverage. The advancement of artificial intelligence, particularly genetic algorithms and neural networks in path planning, offers new solutions to these challenges. Nonetheless, these algorithms often suffer from long learning periods and low efficiency.

Yang and Luo [28] introduced a Biologically Inspired Neural Network (BINN) for Complete Coverage Path Planning (CCPP) that autonomously generates robot paths by leveraging the neural network’s dynamic activity and historical robot locations. This method autonomously plans collision-free paths without requiring pre-existing templates or extensive learning processes, effectively reducing path repetition and ensuring orderly navigation in both familiar and uncharted environments. Additionally, Qiu et al. [29] developed a novel rolling path planning and heuristic search method based on BINN, enhancing its efficacy in uncertain environments. Sun et al. [30] created a multi-AUV full coverage programming method that achieved comprehensive area coverage in underwater settings. These advancements demonstrate the potential of biologically inspired neural networks to provide sophisticated, automated solutions for complex tasks in concrete surface operations, significantly enhancing the precision and operational efficiency of path planning, particularly in environments with complex or irregularly surfaced prefabricated concrete components.

Trajectory planning is essential for robotic operations, particularly for tasks requiring extensive maneuvering such as full coverage smoothing. Standard methods like cubic and quintic polynomial interpolations, while common, present challenges: cubic interpolation can cause sharp changes in angular acceleration, increasing wear on mechanical arms, whereas quintic interpolation, though reducing abrupt movements, may induce larger angular accelerations. To optimize trajectory smoothness and control, a 3-5-3 segmented polynomial interpolation strategy is utilized, effectively blending the benefits of both methods to enhance the efficiency and longevity of robotic systems during complex operations.

This paper focuses on the surface processing technologies of Precast Concrete (PC) components, particularly the smoothing and roughening (brushing) key operations, and addresses the following issues: the development of an integrated robot system for surface smoothing and roughening operations in assembly line production, the design of path planning methods for refined smoothing and roughening, and the feasibility and effectiveness validation of the proposed system and methods through actual prototype experiments. The main contributions of this paper are as follows:

(1) Development of an intelligent robot system for precast concrete components. Compared to solutions from other manufacturers, this system can autonomously complete smoothing, mode switching, and roughening tasks in one go while maintaining a consistent level of smoothness. This significantly enhances work efficiency and reduces labor costs.
(2) Design of refined path planning for smoothing and roughening operations. To achieve refined surface processing, this study designed a path planning method utilizing biologically inspired neural network technology for comprehensive smoothing in scenarios with embedded components. Meanwhile, the A* algorithm is introduced to address the issue of the robot becoming stuck in dead zones during full-coverage operations. Additionally, a 3-5-3 piecewise polynomial interpolation method was employed for trajectory planning to create diverse roughening patterns, ensuring smooth and precise paths.
(3) Validation of the proposed system and methods through actual prototype testing. A scaled-down robot system was developed for the production line, applying the relevant path planning methods. The implementation confirmed the feasibility and efficiency of the proposed system and methods, meeting the demands of actual production.

The remainder of this paper is organized as follows. Section 2 provides a detailed description of the robot’s configuration design and the development of the overall system. In Section 3, we elaborate on the implementation of our refined smoothing and roughening methods. Section 4 presents real robot experiments to validate the feasibility of our system. Finally, in Section 5, we conclude this paper by summarizing the results and discussing potential avenues for future work.

## 2. The Design of the Prototype System

This section is based on the actual situation of the automated production line for PC components, conducting a theoretical design and validation of the configuration of a robot for smoothing and roughening the surfaces of PC components, as well as its processing end-effector. Subsequently, an overall design of the proposed scheme was developed, and a scaled-down experimental system was constructed.

### 2.1. The Conceptual Design of the Robot

#### 2.1.1. Analysis of the Roughening Texture Forming Mechanism

The formation of roughened textures on concrete surfaces through subtractive manufacturing techniques capitalizes on the inherent plasticity of pre-mixed concrete. This process occurs post smoothing operations and prior to the complete hardening of the concrete. It involves the precise manipulation of end-effectors and their trajectories to fabricate intricate surface geometries.

**Figure 1:**
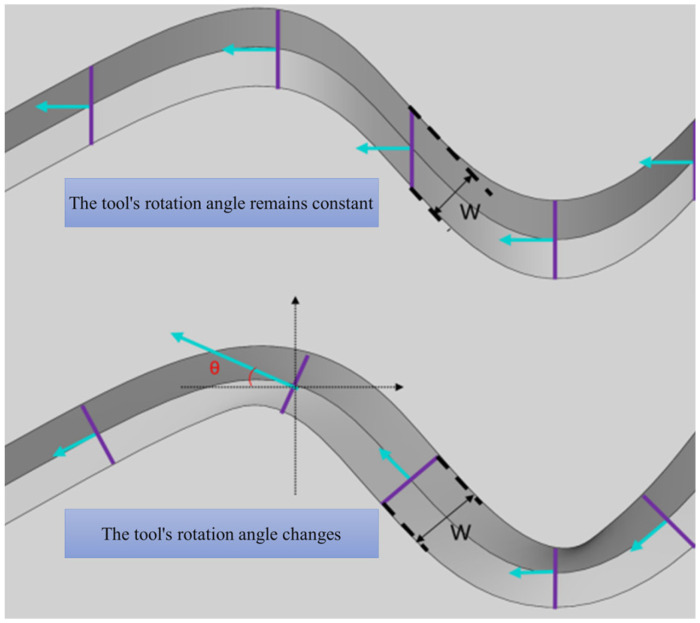
The impact of the vertical axis rotation angle on the texture cross-section.

During the roughening process, the forming procedure consists of a series of precise scraping actions performed by the end-effector along a predefined path on the concrete surface. Key factors influencing the texture shape include the cutting path, texture cross-sectional width W, and cutting depth D. Additionally, as shown in Figure 1, the cross-section of the texture is also affected by the rotational angle θ of the tool around its vertical axis. When the tool’s rotation angle θ remains constant, the width of the texture cross-section is the projection of the tool profile along the path normal; conversely, when the tool plane normal remains parallel to the path tangent, the width of the texture cross-section W equals the width of the tool profile. It is important to note that the texture cross-section referred to here pertains to the contour of the tool as it penetrates below the surface of the concrete.

It is evident that the primary factors in the texture formation of PC components during the subtractive mode include: (1) the toolpath trajectory; (2) the cutting profile; (3) the rotational angle of the tool plane around the vertical axis. This provides a theoretical basis for the subsequent design configuration of the surface smoothing and roughening robot.

#### 2.1.2. The Robot’s Main Body Structure

The robotic system for surface smoothing and roughening of concrete is designed and developed to replace traditional surface smoothing equipment. Existing equipment on Precast Concrete (PC) component production lines almost entirely adopts Cartesian coordinate system robot configurations. Therefore, to minimize alterations to the original layout of the PC component production line, a Cartesian coordinate configuration is utilized for the overall architecture of the surface smoothing and roughening robot [31].

**Figure 2:**
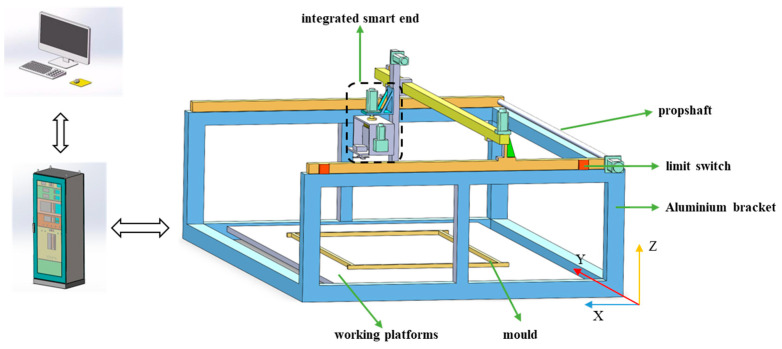
Schematic Diagram of the Overall Structure of the Surface Smoothing and Roughening Robot.

The design of Cartesian coordinate robots is primarily divided into gantry and cantilever configurations. The gantry design, utilizing a dual guideway system, significantly enhances load capacity and structural stability, which is particularly suitable for operations across large spans [31]. For precise surface smoothing and roughening over extended travel distances beyond 3 m, a gantry-type Cartesian coordinate robot is utilized as the principal structure in the automated system for PC components. As depicted in Figure 2, the developed robot features a five-degree-of-freedom design, incorporating three translational degrees (X, Y, and Z) for multidirectional spatial maneuverability. The integrated end-effector includes two rotational degrees of freedom: one around the Z-axis to meet processing needs for paths in different directions, and another around the Y-axis to facilitate tool switching between smoothing and roughening functions, thereby enabling efficient integrated operations.

#### 2.1.3. Multi-Degree of Freedom Integrated Intelligent End-Effector

In the design process of the end-effector, two degrees of freedom are primarily involved. The first degree of freedom is rotation around the Z-axis, which is indispensable for adjusting the direction of the cutting texture trajectory. As described in Section 2.1.1, this allows the end-effector to adjust the texturing style on the concrete surface. Simultaneously, this degree of freedom can be utilized to achieve more uniform smoothing operations. The second degree of freedom involves switching between tools, enabling the end-effector to toggle between smoothing and roughening modes. This switching capability significantly enhances the flexibility and efficiency of operations, allowing a single robotic system to perform multiple tasks concurrently, thereby reducing equipment costs and operational complexity. By integrating these two degrees of freedom, the end-effector not only can finely control texturing through rotational adjustments but also can swiftly change tools to meet different operational demands, effectively combining smoothing and roughening operations into an efficient robotic system.

Additionally, in the design of the end-effector, two critical issues must be considered: (1) how to address the accumulation of excess concrete material during the smoothing process; (2) how to effectively integrate the smoothing and roughening tools.

**Figure 3:**
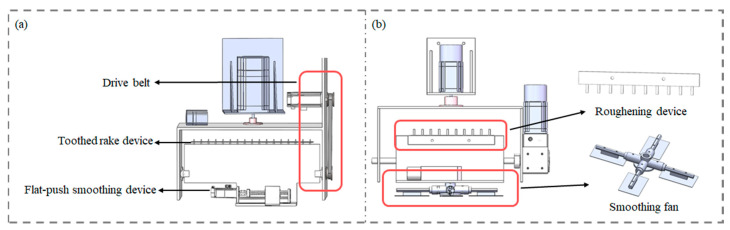
Design scheme of integrated smart end with multiple degrees of freedom: (**a**) the early stage of design; (**b**) the final version of the configuration design.

In the initial design phase, the end-effector configuration, as shown in Figure 3a, utilized a belt drive mechanism for tool switching, employed a flat-push type blade for the smoothing device, and featured a screw motor-driven push rod at the bottom to accomplish the removal of excess material. However, subsequent validation revealed that due to the excessive torque of the drive mechanism, tool switching often resulted in the tools wobbling or stalling. Despite combining flat-pushing with left–right reciprocating motions during the smoothing process, the accumulation of excess material on both sides was still observed. Consequently, in later improvements, as illustrated in Figure 3b, a combination of a gearbox and a stepper motor was employed for the drive mechanism of the smoothing and roughening tools. The gearbox used a 5:1 reduction ratio to increase torque while ensuring speed, facilitating smooth tool switching at the end-effector. The smoothing device adopted a circular fan structure, effectively resolving the issue of material accumulation.

### 2.2. Development of the Robotic Prototype System

The system architecture is bifurcated into hardware and software components. On the hardware side, the motion control card is central to the robotic system’s motion control architecture. It integrates motor drivers and sensors with the upper computer, receiving control commands and processing input from limit switches to manage complex path motions. The card converts joint variables of the robot into pulse signals, facilitating motor operations at set speeds and accelerations, which in turn drive the end-effector to execute smoothing and roughening tasks.

In terms of software, the system is structured into a master control layer, serving as the human–machine interface, and a task layer that delineates critical path points for comprehensive smoothing and roughening paths. The robot’s inverse kinematics translates these path points into joint-specific parameters (position, speed, acceleration). The human–machine interface is segmented into four functional modules: single-axis point motion, robot homing, operation mode selection, and monitoring display, each critical for streamlined robot operations.

#### 2.2.1. Hardware Configuration

**Figure 4:**
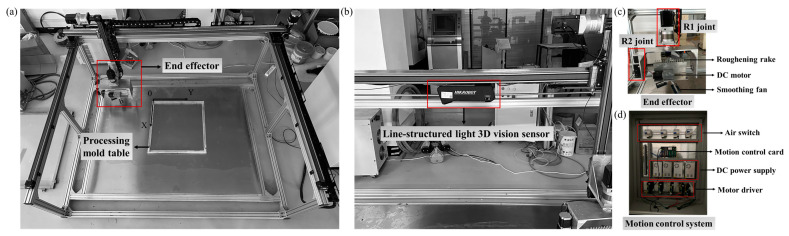
Overall Robotic System’s Hardware Configuration: (**a**) robot body structure; (**b**) line-structured Light 3D Vision Sensor; (**c**) end effector; (**d**) motion control system.

Figure 4 illustrates the prototype of the surface smoothing and roughening robot developed on a reduced scale in our laboratory. The robotic system consists of five parts: (1) photoelectric sensors for limit control; (2) the robot body; (3) the motion control system; (4) the multi-degree of freedom intelligent end-effector; (5) the processing platform; (6) the line-structured light 3D vision sensor.

(1) Robot Mechanical Structure

As shown in Figure 4a, the three-axis translational joints of the surface smoothing and roughening robot are constructed using linear modules with travel distances of 2000 mm, 1500 mm, and 800 mm, respectively, and the processing platform measures 550 mm × 400 mm. The robot’s maximum vertical movement distance is 700 mm, with a maximum speed of 200 mm/s and a maximum load capacity of 30 kg.

The multi-degree of freedom integrated intelligent end-effector is a key component for surface smoothing and roughening operations, consisting of a fan-style smoothing device and a customized roughening tool designed with equidistantly spaced rake teeth. As illustrated in Figure 4c, the overall mechanism is fabricated from aluminum alloy materials, and the smoothing tool is composed of four fan blades made of manganese steel material. Both the smoothing and roughening tools can be manually replaced. The switching device utilizes a servo motor and a gearbox, mounted on the side of the overall tool end, enabling it to drive the tools for flexible switching.

(2) Robot Control System

The motion control system of the robot is composed of a motion control card, drivers, and actuating motors. These components, including the motion control card and drivers, are installed within a control cabinet, the detailed internal layout of which is depicted in Figure 4d. The motor drivers, motion control card, and limit switches are designed to operate at voltages of 220 V, 24 V, and 24 V, respectively. Consequently, in the process of selecting and arranging the power modules, both the first and third layers of the cabinet are configured to include a row of air switches and a row of 24 V DC power modules. The various components and power modules are isolated from each other using trunking made from insulating and flame-retardant materials, which serve to prevent damage to the overall system due to potential component failure. All electrical connections within the system are made via terminal blocks, which not only increase the reliability and efficiency of the system but also simplify the processes of installation and maintenance, thus facilitating future component replacement and servicing.

Industrial robots commonly employ embedded motion controllers, Programmable Logic Controllers (PLCs), and motion control cards as primary controllers. Compared to PLCs and embedded controllers, motion control cards use standard PCs, enhancing integration with machine vision systems which is advantageous for vision inspection tasks in smoothing and roughening robots. Due to the complexity of freeform curves required for surface texture processing in three-dimensional spaces, this paper selects the Motiontek NMC5800 motion control card for the robot’s control system. This model supports up to 8 axes of continuous trajectory motion per card, expandable up to 64 axes through cascading, facilitating future system expansions. It also accepts inputs for 8 home, positive limit, and negative limit signals, and offers 24 V output voltage with grounding protection. The stepper motor operates via the “pulse + direction” method, utilizing API functions from Motiontek, and a personal computer, enabling precise execution of various motion commands such as homing, point-to-point movement, and continuous interpolation.

Given the precision requirements for PC component fabrication are within 1–3 mm, which do not fall into the high-precision category, and the processing speed is limited to 50 mm/s, scenarios involving high-speed movements are not anticipated. Therefore, there is no need to consider the decrease in stepper motor torque during high-speed operations. Consequently, three sets of two-phase, four-wire 86 stepper motors have been chosen to drive the overall motion of the machine. For the vertical direction, motors equipped with electromagnetic brakes are selected to achieve power-off self-locking, enhancing the safety of the robot system. Specifically, the motors for the X and Y axis joints are Leadshine 86CM85-BZ model stepper motors, which have a rated torque of 8.5 N⋅m. For the Z-axis joint, the Leadshine 86CM45-BZ model stepper brake motor is selected, with a rated torque of 4.5 N⋅m, paired with an M860D motor driver. The R1 joint utilizes an 86 stepper motor, and the R2 joint employs a 57 stepper motor, both equipped with a 5:1 gearbox for driving, fulfilling the requirements for precise positioning. The motors for the X, Y, Z, R1, and R2 joints are all manufactured by Leadshine in Guangdong, China.

The limit switches employ FM-SX67 series photoelectric sensors with an NPN output mode, manufactured by Zhiman Electronics Co., Ltd. in Foshan, Guangdong Province, China. When a metal piece enters the interior of the photoelectric sensor, it outputs a high signal, causing the indicator light on the motion control card to turn off and halting the motion of the single axis. During installation, the black control wires of the limit switches are commonly connected to the negative limit (EL-) and home limit (ORG) points on the motion control card.

(3) Line-structured Light 3D Vision Sensor

As shown in Figure 4b, the experimental platform employs the Hikvision’s MV-DL2025-04H-H line-structured light 3D vision sensor from Hangzhou, Zhejiang, China, for the collection of three-dimensional point clouds. Based on the acquired point clouds, the platform completes the presentation of concrete surface smoothing results. Its scanning frame rate is 600 fps, the laser frequency is 650 nm, with near and far field ranges of 1000 mm and 2600 mm, respectively, and a measurement height range within 1500 mm.

In scenarios where line-structured light 3D vision sensors are used, a base plane typically exists upon which all test objects are placed. In the smoothing and deburring robot system, this base plane is the model stage. The line-structured light 3D vision sensor is mounted at the central position of the robot’s Y-axis and collects point clouds vertically downwards from the base plane. Within the line-structured light 3D sensor, there are two coordinate systems: one is the camera coordinate system established on the 2D camera lens, and the other is the sensor coordinate system established on the plane formed by the laser as it propagates in space. The origin of the sensor coordinate system is the intersection of the laser source’s central line with the base plane, which requires adjustments based on the actual base plane in the working scene. The 3DMVS2.1.0 software provided by Hikvision offers functionality for calibrating the transformation matrix, with the calibration steps as follows:

(1) Turn off the laser and HDR enable, use the image acquisition mode, and additionally use an infrared light source to illuminate the calibration board. Adjust the exposure time to between 5000 μs and 10,000 μs;
(2) Move the 3D vision sensor to capture images of the calibration board at two different positions;
(3) Enter the calibration board’s chess grid length and width parameters, the number of chess grid parameters, and the height of the 3D sensor relative to the base plane. Click the start calibration button to compute the transformation matrix.

After calibration, to accurately obtain the precision of concrete surface inspection, the detection algorithm is defined. Considering development efficiency, detection speed, and the regular geometric features of the module itself, this paper adopts a 3D vision module detection algorithm based on geometric features. The overall algorithm process is divided into two major parts. The first part is processed in three-dimensional space, starting with a pass-through filter to determine the approximate ROI area, followed by statistical filtering to remove outliers, then Gaussian filtering for point cloud smoothing, and subsequently segmenting the point cloud using a region growing method, followed by using the RANSAC algorithm to identify and filter non-planar point clouds. The second part involves processing after projecting the planar point clouds into two-dimensional depth images, beginning with median filtering and morphological closing operations for preprocessing the depth images, then by extracting the dimensions of the minimum bounding rectangle to judge the point clouds of various rectangular sections of the prefabricated parts, followed by matching the sections of the prefabricated parts, and finally calculating the module’s position and orientation information.

#### 2.2.2. Software Configuration

**Figure 5:**
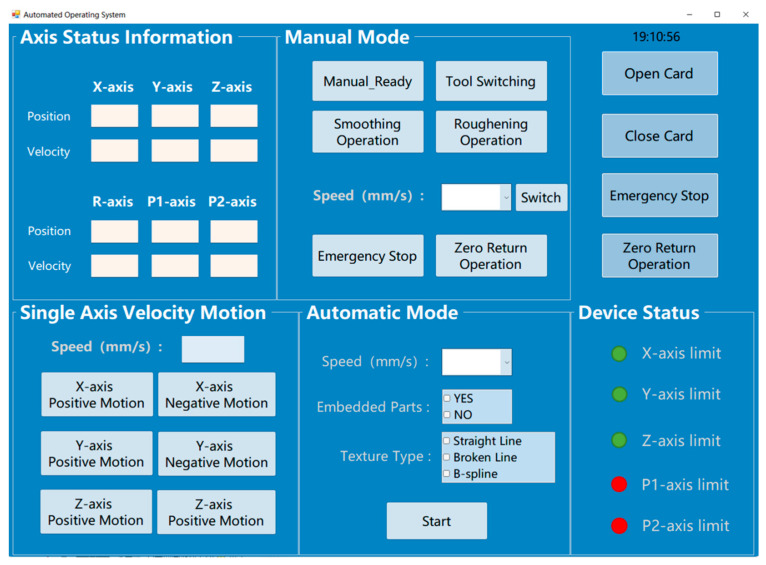
Upper computer interface.

This paper presents the design of an upper computer software system capable of performing smoothing, roughening, and automatic tool switching operations, as depicted in Figure 5. Developed for the Windows operating system, the software interface encompasses the following key functionalities: (1) automated processing for smoothing and roughening; (2) single-axis point motion control; (3) homing to the origin; (4) display of the robot body’s joint motion information and processing path information; (5) display of limit switch status. The software system adopts an intuitive button-based design, allowing users to flexibly control the robot and plan operations based on current needs. During single-axis point motion, users can easily set parameters such as speed and acceleration for each axis to facilitate motor fault testing. In surface processing operations, the software enables not only step-by-step actions but also automated processing for smoothing, tool switching, and roughening. Users can clearly observe the robot’s current motion path points through the position and running speed of each axis. Additionally, operators can monitor the status of limit switches for each axis in real time, ensuring that the machine does not exceed its operational range during operation, thereby safeguarding the equipment, workpieces, and operator safety. Moreover, the interface’s upper right corner features functions for opening and closing the motion control card. An emergency stop function has been added to each functional area to prevent motor malfunctions from causing looseness in the linear modules.

#### 2.2.3. Repeat Positioning Accuracy Test of End-Effector

**Figure 6:**
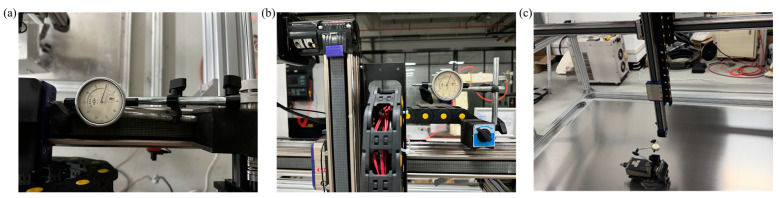
The testing process for repeat positioning accuracy: (**a**) X-axis; (**b**) Y-axis; (**c**) Z-axis.

After the overall prototype and control system design are completed, it is necessary to test the positioning accuracy of the robot’s entire mechanical structure, which involves measuring the discrepancy between the set values and the actual displacement values of the robot’s end-effector. The specific method involves setting the motion mode in the motion control card to position mode. The repeatability of the positioning accuracy for the three motion mechanisms is tested using a magnetic base combined with a dial indicator with a precision of 0.01 mm, as illustrated in Figure 6. The initial dial indicator readings for each axis are recorded, followed by recordings of the dial indicator after four sets of 200 mm back-and-forth movements, totaling five readings, to calculate the error. The final error results are presented in Table 1.

Based on the test data, the repeat positioning accuracy of these three axes is within 0.5 mm, which meets the precision requirements of the robot system during the smoothing and roughening operations.

#### 2.2.4. Calibration and Positioning Accuracy Testing of Vision Sensors

In Hikvision’s 3DMVS2.1.0 software, following the calibration method for the transformation matrix between the camera coordinate system and the sensor coordinate system, images of the calibration board are captured at two different positions. Calibration board parameters are then inputted to calibrate and obtain the transformation matrix between the two-dimensional camera coordinate system and the sensor coordinate system. The calibration results for the transformation matrix are as follows:

(1) $$T=\left[\begin{array}{cccc} -0.999997 & -0.00186962 & -0.00152059 & 111.798\\ -0.00133087 & 0.954465 & -0.29832 & 269.656\\ 0.00200909 & -0.298317 & -0.954465 & 891.506 \end{array}\right]$$

Subsequently, the detection accuracy is tested on the experimental platform. After the smoothing process, the four edge molds of the concrete precast parts are scanned to detect and locate the positions of each edge mold module in the point cloud. The detection results are shown in Table 2.

Analysis of the module detection experimental results indicates that the positional error of the algorithm is within 1 mm, which meets the requirements for surface inspection of concrete precast components during the smoothing process.

## 3. Planning Method for Processing

In this section, we introduce our method for planning the smoothing and roughening operations, specifically including a full-coverage smoothing path planning method and a roughening operation planning method for curved textures. Additionally, in the robot’s joint space, we present a 3-5-3 piecewise trajectory planning method.

### 3.1. Smoothing Path Planning Method

**Figure 7:**
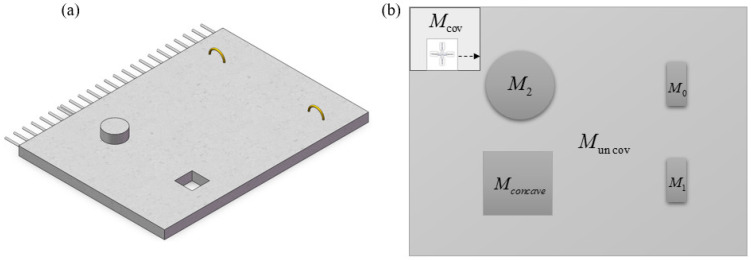
The schematic diagram of the surface conditions of prefabricated components: (**a**) a three-dimensional schematic diagram; (**b**) a two-dimensional surface schematic diagram.

The surface of precast concrete components can be conceptualized as a rectangular area. As illustrated in Figure 7, the entire task area G for smoothing operations can be subdivided into the smoothed area $M_{\mathrm{cov}}$, uncovered area $M_{un\mathrm{cov}}$, hooks $M_{0}\backslash M_{1}$ and pipe $M_{2}$, as well as reserved hole area $M_{concave}$. The focus of this section is to address the smoothing operation problem by acquiring obstacle information on the concrete surface based on the environmental conditions, establishing a surface environment model, and devising a path that covers a wide area, promptly avoids obstacles, and minimizes path length.

We can uniformly define the smoothing operation as the CCPP problem (Complete Coverage Path Planning). The key to the CCPP problem lies in how to traverse the maximum extent of the workspace area excluding obstacles and effectively avoid all obstacles. The problem of complete coverage path planning for smoothing the surface of concrete with obstacles can be formalized through a triple $\left(G,M_{obstacle},P\right)$, where:

(1) *G =* (*M*,*E*) represents the model of the concrete surface to be covered, where M is the set of vertices in the graph corresponding to all points on the concrete surface (including hooks $M_{0}\backslash M_{1}$, pipe $M_{2}$, and the non-obstacle area $M_{uncov}$), and E is the set of edges representing the paths along which the smoothing robot can move on the surface of the prefabricated component.
(2) $M_{obstacle}\subseteq M$ is a subset of the graph G, representing the vertices occupied by obstacles on the concrete surface. These vertices correspond to non-smoothable areas, including protruding embedded components $M_{convex}\left(M_{1},M_{2}\right)$ and reserved hole positions $M_{concave}$.
(3) $P=\left(P_{1},P_{2},P_{3},\ldots ,P_{n}\right)$ is a sequence of coordinate points representing the movement path of the smoothing robot. This path should satisfy the following conditions: the starting point $p_{1}$ and the ending point $p_{n}$ are predefined start and end points for smoothing, each pair of consecutive vertices $\left(p_{i},p_{i+1}\right)$ in the path must belong to E, and the path must cover all vertices in $M\backslash M_{obstacle}$ at least once, ultimately achieving complete coverage smoothing of non-obstacle areas.

The common full-coverage traversal methods include the “back-and-forth” method and the “spiral” method. Due to the excessive viscosity of the concrete surface and issues with residue accumulation, the “spiral” method for full coverage can lead to the phenomenon of concrete residue gathering inward. Meanwhile, compared to the “spiral” method, the “back-and-forth” walking pattern can generate shorter paths for area transitions. Therefore, this paper adopts the “back-and-forth” full-coverage smoothing method.

#### 3.1.1. Without Embedded Parts

**Figure 8:**
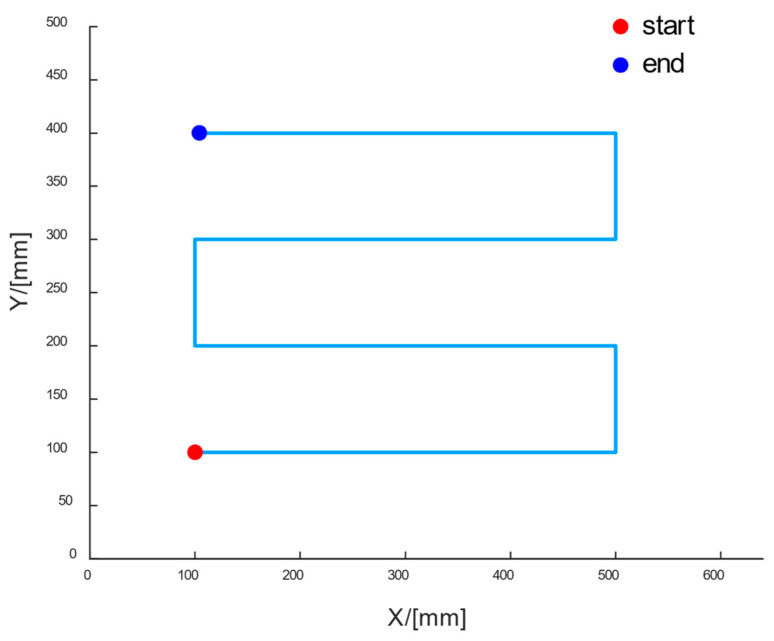
Smoothing Operation Path Planning Diagram.

During the smoothing operation, when there are no obstacles on the surface, a point-to-point path planning method is employed. As shown in Figure 8, at the initial state, the robot’s end effector for smoothing is located at (100, 100) in the coordinate system of the operation mold. When smoothing in the positive direction of the X-axis, it reaches the edge of the component at (500, 100). At this point, it moves in the positive direction of the Y-axis to (500, 200) and then starts a new round of smoothing work in the opposite direction. This process is repeated until the final smoothing operation is completed.

#### 3.1.2. With Embedded Parts

When precast parts have pipes, hooks, or other embedded parts on their surface, point-to-point path planning is no longer applicable. In such cases, we need to model and analyze the environment map of the concrete surface, calibrate the location of the embedded parts, and use a biologically inspired neural network to carry out full-coverage path planning on the concrete surface. The biologically inspired neural network model can comprehensively consider all factors in the environment, such as obstacles, the size and shape of target areas, and possible paths. At the same time, it can integrate and adapt well to two-dimensional grids during the process of environmental modeling.

(1) Grid Map Building and Neural Network Representation

**Figure 9:**
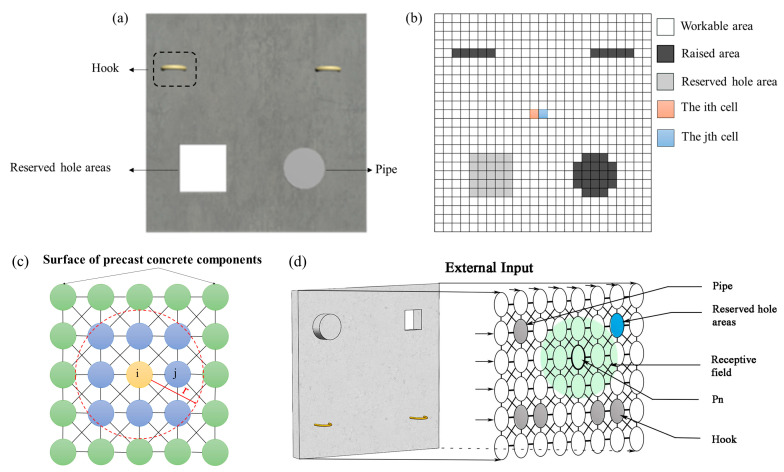
Environmental conditions on the surface of precast concrete components: (**a**) 3D maps of precast surfaces; (**b**) grid map of the precast part surface; (**c**) 2D neural network model; (**d**) the discrete topological organization diagram.

In conventional grid maps, the obstacle areas and feasible workspace for robotic operations are represented by black and white grids, respectively, indicating the regions where obstacles are located and where operations can be conducted. However, on the surface of precast concrete components, there are mainly raised areas such as hooks that cannot be reached for smoothing. Additionally, although reserved hole areas can be traversed, they are considered non-functional workspace areas that need to be avoided during operation planning. Therefore, during programming implementation, it is necessary to use a two-dimensional array for representation. The values in the array indicate the status of each grid: 1 represents feasible workspace, −2 represents raised portions, and −1 represents reserved hole areas. The indices of the two-dimensional array correspond to the specific positions of the grids in the two-dimensional space. The specific model is illustrated in Figure 9b.

In the grid map, each cell with its own neural activity is considered as a neuron, and each neuron has lateral connections only to neighboring neurons to form a neural network. As shown in Figure 9c, a 2D neural network is built on the grid map, the *i*th cell and *j*th cell in Figure 9b correspond to the *i*th neuron and *j*th neuron in Figure 9c, respectively. In Figure 9c, the receptive field of the *i*th neuron is represented by a circle with radius *r*, indicating that the neuron responds only to the stimulus within its receptive field.

(2) Biologically Inspired Neural Network Algorithm Model

A computational membrane model in a biological neural system was proposed by Hodgkin and Huxley [32]. Yang and Luo [28] improved the shunting equations based on this model, established a dynamic neural network structure, and applied it to the CCPP of the cleaning robot.

In the biologically inspired neural network, the environment is defined as a neural network topological state space, placed within a Cartesian coordinate system of the environmental grid map model. Additionally, in the bio-inspired neural network model, a shunting equation is defined to guide the robot’s adjustment of the current state based on the activity values in the neural network’s topological space. As shown in Figure 9d, the neural network system on the surface of precast concrete parts is a discrete topological organization diagram, with only local lateral connections between adjacent neurons. In this way, the neural activity in the movable area can guide the movement of the smoothing and leveling robot in the state space, while the embedded parts only have an effect within a local range to avoid collisions. The neural activity $x_{i}$ of the *i*th neuron on the surface of a precast concrete part can be characterized by the following equation:

(2) $$\frac{dx_{i}}{dt}=-Ax_{i}+(B-x_{i})({\left[I_{i}\right]}^{+}+\sum_{j=1}^{k}w_{ij}{\left[x_{j}\right]}^{+})-(D+x_{i}){\left[I_{i}\right]}^{-}$$

where $x_{j}$ is the neural activity of the *j*th neuron, and *k* represents the number of neurons which share connections with the *i*th neuron in the receptive field. The terms ${\left[I_{i}\right]}^{+}+\sum_{j=1}^{k}w_{ij}{\left[x_{j}\right]}^{+}$ and ${\left[I_{i}\right]}^{-}$ are the external excitatory and inhibitory inputs of the neuron. Functions ${\left[f\right]}^{+}$ and ${\left[f\right]}^{-}$ are defined as ${\left[f\right]}^{+}=\max\left\{f,0\right\}$ and ${\left[f\right]}^{-}=\max\left\{-f,0\right\}$, respectively. The external input $I_{i}$ is defined as

(3) $$I_{i}=\{\begin{array}{ll} E & \mathrm{uncovered}\;\mathrm{area}\\ \begin{array}{l} -2E\\ -E \end{array} & \begin{array}{l} \mathrm{hooks}\;\mathrm{and}\;\mathrm{pipeline}\;\mathrm{area}\\ \mathrm{reserved}\;\mathrm{hole}\;\mathrm{position}\;\mathrm{area} \end{array}\\ 0 & \mathrm{otherwise} \end{array}$$

where *E* represents the value of external excitatory input, while −*E* and −2*E* represent the values of external inhibitory inputs. Typically, −2*E* indicates obstacles such as protruding hooks and pipes, hence exerting stronger inhibitory effects. The value $w_{ij}$ represents the weight of the connection between the *i*th and *j*th neurons, which is defined as

(4) $$f(a)=\{\begin{array}{ll} \mu /a & \mathrm{if}(0<a<r_{0})\\ 0 & \mathrm{if}(a\geq r_{0}) \end{array}$$

where a=|i−j|, if $a\leq r_{0}$ then f(a)=0, if $0<a<r_{0}$ then f(a)=μ/a, and μ is a positive constant.

From the shunt equation, it can be seen that a positive value of neuronal activity is a positive excitation and is able to spread globally over time, and a negative value of neuronal activity can only affect the surrounding neurons. The activity values of the obstacle cells and the target cells correspond to the troughs and peaks, and the core of the biologically inspired neural network path planning algorithm is that the positive activity values of the target grid diffuse in the global space, motivating it to move towards the grid cells at the center of the diffusion, and the obstacle cells are only able to act locally due to their negative activity values, repelling the robot’s approach.

The biologically inspired neural network algorithm can accomplish both point-to-point and full-coverage path planning, with the robot generating point-to-point paths as

(5) $$p_{n}⇐x_{p_{n}}=\max\left\{x_{j},j=1,2,\ldots ,k\right\}$$

where the path generation process is as follows: the robot selects the neuron grid with the largest activity value among neighboring neurons at the current position as the next position, and after reaching the next position, the next position becomes the new current position, and so on, until it reaches the target point grid.

Robots generate full coverage paths for

(6) $$p_{n}⇐x_{p_{n}}=\max\left\{x_{j}+cy_{j},j=1,2,\ldots ,k\right\}$$

where c is a positive constant representing the choice of orientation weights, $y_{j}$ is a function related to the robot’s last location $p_{p}$, current location $p_{c}$, and next location $p_{j}$, and the $y_{j}$ function is defined as

(7) $$y_{j}=1-\frac{\Delta \theta_{j}}{\pi }$$

where $\Delta \theta_{j}\in [0,\pi ]$ represents the angle between the current direction and the next direction.

The CCPP algorithm based on BINN operates without the need for any prior information or manual intervention. Additionally, the neural network functions as a stable system. Yang and Meng [33] rigorously proved the stability and convergence of the neural network model using the Lyapunov stability theory.

(3) Deadlock Escape

During the CCPP, the robot may be trapped into the deadlocks by its own path. In the model based on BINN, this issue is often determined by the relative high or low values of neuronal activity. Specifically, when all neighboring neurons and the central neuron exhibit equally minimal neural activities, the robot may become trapped in several covered path points because it cannot select an appropriate path point through Equations (6) and (7). Therefore, without additional escape strategies, the algorithm will struggle to effectively guide the robot out of such dead zones and towards nearby uncovered areas to continue the task. This scenario not only affects the robot’s work efficiency but may also lead to incomplete task execution.

Given the known environmental map information, the point-to-point path planning method will be employed, utilizing the A* algorithm to facilitate the quick escape of the smoothing robot from dead zones and to continue with subsequent tasks. The A* algorithm can effectively integrate with grid map environments, aiding the robot in quickly finding the optimal path to escape from dead zones. The heuristic function of the A* algorithm is represented as follows:

(8) f(n)=g(n)+h(n)

where, in this context, f(n) is the estimated cost from the initial state, via state n, to the target state; g(n) is the actual cost in the state space from the initial state to state n; h(n) is the estimated cost of the best path from state n to the target state.

In this process, the accuracy of the position estimation is crucial. Designing a reasonable cost function is key to ensuring that path planning effectively integrates with the actual map environment. Currently, mainstream cost functions include Manhattan distance and Euclidean distance. Given that the number of grid cells within dead zones is relatively low in the algorithm studied in this paper, the Euclidean distance function is chosen as the primary method for calculating the escape cost in order to enhance the accuracy of the escape path. Although this method may slightly increase the search burden, it can more accurately reflect the real layout of the environment.

(4) Simulation Results and Comparison

**Figure 10:**
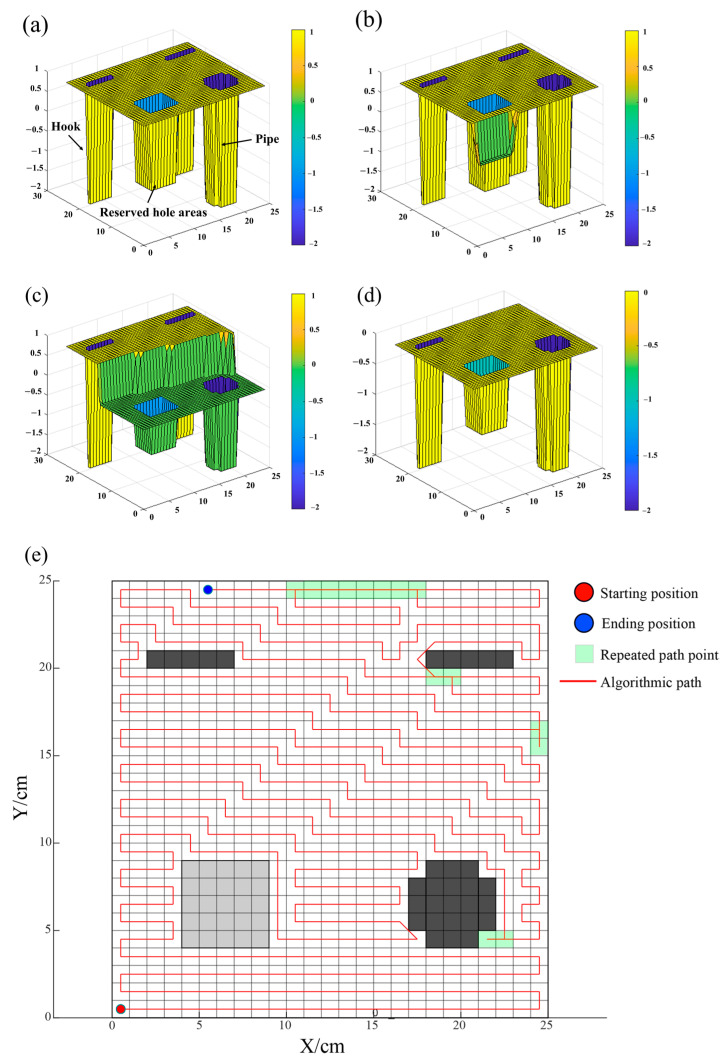
The final result of the path planning: (**a**) initial state activity value; (**b**) mid-time activity value; (**c**) post-obstacle activity value; (**d**) post-CCPP operation activity value; (**e**) two-dimensional path diagram.

When obstacles such as pipes, hooks, and reserved holes are present on the surface to be smoothed, the final effect is verified using the BINN algorithm. All environmental information is also known, with the grid size set at 25 × 25, and each grid measuring 1 cm in length. With A = 10, B = 1, D = 1, E = 1, u = 1, and r_0_ = 2, the smoothing robot can only search for information in eight neurons within its vicinity. The task is completed when all areas of the concrete surface that need work are covered. The final result of the path planning is shown in Figure 10.

From Figure 10a, the path provided by the BINN algorithm starts at the coordinate point (0, 0), entering the full-coverage smoothing operation phase. At this initial moment, hooks and pipes are located at trough positions, reserved holes are between troughs and peaks, and the concrete areas to be worked on are at peak positions, conforming to the initial state settings of the BINN algorithm. During the back-and-forth operation, the robot first encounters embedded pipes, followed by reserved holes, and during this period, the current activity values calculated by the BINN algorithm facilitate the back-and-forth full-coverage operation. Figure 10e shows the full-coverage path map for the smoothing operation, where light gray represents reserved holes and dark gray represents protruding embedded components. When passing through the embedded pipe at coordinate (21, 4), the BINN algorithm reveals that the activity values of surrounding neurons are less than those at the current location, causing the algorithm to enter a deadlock. By recalculating using the A* algorithm, the deadlock is successfully escaped. Similarly, deadlocks at (21, 4), (24, 15), (20, 19), and (18, 24) are escaped using the A* algorithm with good results. Overall, the optimal final path is 568 cm, the total path length is 581 cm, with a path redundancy rate of 2.3%, and a smoothing coverage of 100%, effectively completing the surface smoothing operations on the precast pieces.

**Figure 11:**
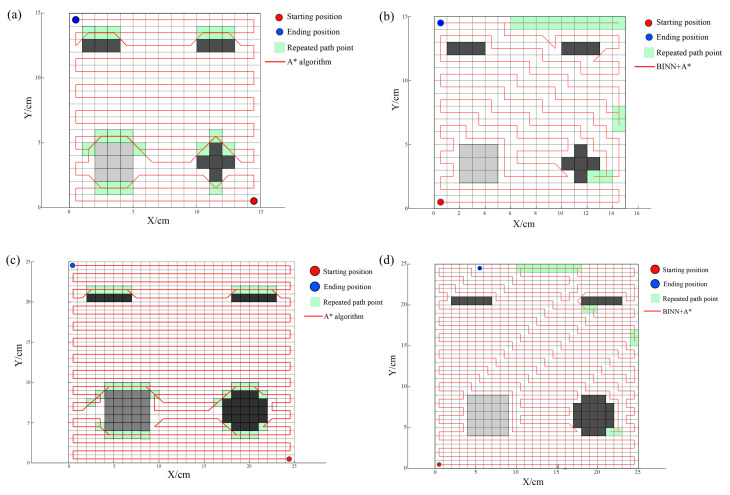
The full-coverage path planning maps under the 15 × 15 and 25 × 25 grid maps: (**a**) A* algorithm on a 15 × 15 grid map; (**b**) BINN + A* algorithm on a 15 × 15 grid map; (**c**) A* algorithm on a 25 × 25 grid map; (**d**) BINN + A* algorithm on a 25 × 25 grid map.

To better validate the superior performance of the smoothing operation methods, this paper’s proposed path planning method will be compared with the traditional A* algorithm path planning. Comparative experiments of different full-coverage algorithms will be conducted on both 15 × 15 and 25 × 25 grid maps. The main focus will be on three key performance indicators: coverage rate, path length, and path redundancy rate. Figure 11 shows the full-coverage path planning maps under the 15 × 15 and 25 × 25 grid maps.

Table 3 presents the final comparative results between the two algorithms. Based on the simulation analysis, it is evident that the method proposed in this paper, which combines the BINN-based complete coverage path planning algorithm with the A* escape algorithm, is more suitable for addressing the comprehensive coverage smoothing operations on complex concrete surfaces, achieving comprehensive and efficient coverage. Compared to the traditional A* algorithm, this combined approach demonstrates superior performance across key performance metrics such as coverage rate, path length, and path repetition rate. Specifically, on a 25 × 25 grid map, the algorithm’s performance is particularly remarkable. While maintaining the overall coverage rate nearly constant, the path length is shortened by 24 cm compared to the traditional A* algorithm, and the overall path repetition rate is reduced by 3.3%. Additionally, the integration of the A* algorithm enhances the precision of robot maneuvers in dead zone escape and obstacle avoidance, thereby optimizing the smoothness and rationality of global path planning.

### 3.2. Roughening Processing Path Planning Method

When roughening the surface of concrete to display a variety of roughening patterns, we can use different texture forms such as straight, polyline, and curve types for concrete surface roughening. It is noteworthy that the first two types of roughening patterns can be accomplished with point-to-point path planning. However, curvilinear roughening patterns cannot be completed through point-to-point path planning. In such cases, we can describe the roughening information on the concrete surface using B-spline curve fitting. This type of curve offers advantages such as smooth inflection points and small displacement change rates. The general expression of the B-spline curve equation is as follows [34]:

(9) $$p(u)=\sum_{i=0}^{n}d_{i}N_{i,k}(u)$$

(10) $$\{\begin{array}{l} N_{i,0}(u)=\{\begin{array}{l} 1,\left(u_{i}\leq u\leq u_{i+1}\right)\\ 0,\;\mathrm{others}\; \end{array}\\ N_{i,k}(u)=\frac{u-u_{i}}{u_{i+k}-u_{i}}N_{i,k-1}(u)+\frac{u_{i+k+1}-u}{u_{i+k+1}-u_{i+1}}N_{i+1,k-1}(u)\\ \;define\;\frac{0}{0}=0 \end{array}$$

where $d_{i}$ denotes the control vertex of the B-spline curve, $N_{i,k}(u)$ denotes the basis function of the spline function, and *k* denotes the number of curves. The expression of the basis function $N_{i,k}(u)$ based on the recursive formula is shown in (10). In this paper, set *k* = 3. The solution of $N_{i,k}(u)$ can be obtained by a recursive formula after we determine *k* + 2 nodes such as $u_{i},u_{i+1},\cdots ,u_{i+k+1}$. If there are m − 1 control points, the node vector can be written as $U=\left[u_{1},u_{2},u_{3},\cdots ,u_{m+k}\right]$. Define a time series $T=[t_{1},t_{1},\cdots ,t_{m-1}]$ where the time interval $\Delta t=t_{i+1}-t_{i}$ is proportional to the distance between the control points. The normalized time series produces the following internal nodes [34]:

(11) $$u_{i+k+1}=u_{i+k}+\frac{t_{i+1}-t_{i}}{t},i=1,2,\cdots ,m-2$$

Meanwhile, the r-order derivative of the cubic non-uniform B-spline curve can be calculated based on Equation (12) as

(12) $$\{\begin{array}{l} p^{r}(u)=\sum_{j=i-3+r}^{i}d_{j}^{r}N_{j,3-r}(u),u\in \left[u_{i},u_{i+1}\right]\\ d_{j}^{l}=\{\begin{array}{l} d_{j},l=0\\ (4-l)\frac{d_{j}^{l-1}-d_{j-1}^{l-1}}{u_{j+4-l}-u_{j}},l=1,2,\cdots ,r;j=i-3+l,\cdots i \end{array} \end{array}$$

where, according to the known coordinates of the control points, the number of curves k and the basis function, a trajectory based on the B-spline curve can be determined. At the same time, the information about the derivatives of the B-spline can also determine the position, velocity, and acceleration values of the joints at any interpolation value, which can complete the automated hair pulling operation of the robot.

### 3.3. A Trajectory Planning Method for Robot Joint

For full-coverage smoothing path planning as well as straight line and broken line roughening path planning, we use a point-to-point planning method. These discrete points only contain information about the X, Y, and Z position coordinates of each point, and we also need to carry out trajectory planning in the joint space to form curves of position, velocity, and acceleration over time. Getting the relevant velocity and acceleration information will ensure that the interpolation algorithm of the motion control card generates a continuous and smooth motion path. The normal interpolation algorithms are mostly cubic polynomial interpolation and quintic polynomial interpolation. During the “round-trip” full-coverage smoothing process, each round-trip involves an emergency stop and start of one of the robot’s axes. If a cubic polynomial interpolation is used, it will result in making the acceleration at the start and end points change abruptly, which will increase the wear and tear of the arm mechanism and shorten the service life of the arm. Although fifth-degree polynomial interpolation prevents abrupt changes in angular acceleration, the use of fifth-degree polynomial interpolation to plan trajectories produces large accelerations. Between the advantages of both, we will use 3-5-3 segmented polynomial interpolation for trajectory planning in the robot joint space.

For joint trajectories consisting of N segments, the planning time for the robotic arm is first normalized [35] as

(13) $$t=\frac{T-T_{i-1}}{T_{i}-T_{i-1}},t\in [0,1]$$

where *t* is the normalized time variable, *T* is the time actually used by the joint, and $T_{i}$ is the time actually used by the end of the robotic arm from the start point to the termination point of the *i*th trajectory. Each segment can be represented as a function of the curve about time *t* [36] as

(14) $$\{\begin{array}{c} \theta_{1}(t)=a_{10}+a_{11}t+a_{12}t^{2}+a_{13}t^{3}\\ \theta_{2}(t)=a_{20}+a_{21}t+a_{22}t^{2}+a_{23}t^{3}+a_{24}t^{4}+a_{25}t^{5}\\ \theta_{3}(t)=a_{30}+a_{31}t+a_{32}t^{2}+a_{33}t^{3} \end{array}$$

We are using 3-5-3 segmented polynomial interpolation in order to make the planned trajectory continuous and smooth, thus requiring constraints on displacement, velocity, and acceleration as shown below [36]:

(15) $$\{\begin{array}{c} \theta_{1}(0)=\theta_{0},\theta_{2}(0)=\theta_{1}(1)\\ \theta_{3}(0)=\theta_{2}(1),\theta_{3}(1)=\theta_{f}\\ {\dot{\theta }}_{1}(0)={\dot{\theta }}_{0},{\dot{\theta }}_{2}(0)={\dot{\theta }}_{1}(1)\\ {\dot{\theta }}_{3}(0)={\dot{\theta }}_{2}(1),{\dot{\theta }}_{3}(1)={\dot{\theta }}_{f}\\ {\ddot{\theta }}_{1}(0)={\ddot{\theta }}_{0},{\ddot{\theta }}_{2}(0)={\ddot{\theta }}_{1}(1)\\ {\ddot{\theta }}_{3}(0)={\ddot{\theta }}_{2}(1),{\ddot{\theta }}_{3}(1)={\ddot{\theta }}_{f} \end{array}$$

According to Equations (14) and (15), the relationship between displacement, velocity, and acceleration of each joint can be obtained to achieve the final 3-5-3 segmented polynomial interpolated trajectory planning.

## 4. Experimental Results

In this section, several real robot experiments were conducted to test the system’s performance in smoothing and roughening. During the smoothing phase, two experiments were carried out, one without embedded objects and one with embedded objects. In the roughening phase, three sets of roughening experiments were performed for different operational trajectories. Ultimately, the feasibility and effectiveness of the robotic system were validated.

### 4.1. Experimental Setup

Based on our research into the actual production line for precast components, this paper will use 1.5 kg of water, 4 kg of cement, and 2 kg of fine sand as our experimental materials, which are mixed well to make the concrete workpieces. Additionally, the size of the concrete mold is 600 mm × 600 mm × 20 mm (length × width × height). The environmental temperature is set between 12–24 °C, with a relative humidity of 60–72%. The specific steps in the actual experiment process are as follows:

(1) Zeroing of the overall mechanism. Before starting all operations, it is necessary to reset the origin, i.e., return to the origin of the current terrestrial coordinate system. This not only verifies the feasibility of the trajectory planning but also provides a guarantee for the planning of subsequent operation points.
(2) Surface smoothing operation. After the concrete formwork and pouring are completed, the upper computer controls the motor to complete the robot’s smoothing operation within the mold.
(3) Switching of operation tools. After the smoothing operation is finished, the robot’s end mechanism is raised to an appropriate position and moved to a safe position on the side of the concrete mold. The final tool switching process is then completed through the coordination of the motor and the integrated end, preparing for the subsequent roughening operation.
(4) Roughening operation. After the tool switching is completed, the concrete is allowed to sit for a period before starting the roughening operation. This is to prevent the surface viscosity from being too high, which could lead to the adhesion of concrete residues on the cutter head and reduce the smoothness of the textured surface.

### 4.2. Actual Testing of The Smoothing Operations

In this section, we employ the method proposed in Section 3.1 to conduct smoothing experiments under two conditions: with and without embedded objects (each experiment was repeated five times). Trajectories for the end-effector were generated based on waypoint information, and relevant experimental data were input into the motion control card for real experimental validation. By scanning the concrete surface with a scanner and processing the data for visualization, we quantitatively analyzed the smoothness of the concrete surface, thereby validating the feasibility of the smoothing operations proposed by our system.

To quantitatively assess the robot’s smoothing effects, the following indicators were used:

(1) Surface Height Mean, $\bar{Z}$, defined as the average Z-coordinate of all the highest points on the concrete surface:
  (16) $$\bar{Z}=\frac{z_{1}+z_{2}+z_{3}+\cdots +z_{n}}{n}$$
  where $z_{i}\left(i=1,2,3,\cdots ,n\right)$ represents the Z-coordinate of the highest layer in the point cloud data. Ideally, $\bar{Z}=Z_{mould}=20\;\mathrm{mm}$, and we defined the smoothing quality as successful if it fell within the range of 19.5 mm to 21 mm.
(2) Standard Deviation, defined as the dispersion of the concrete surface heights:

(17) $$\sigma =\sqrt{\frac{\sum_{i=1}^{n}{\left(z_{i}-\bar{Z}\right)}^{2}}{n}}$$

When σ≤1, we considered the concrete surface to be smooth, indicating a good smoothing effect.

#### 4.2.1. Without Embedded Parts

**Figure 12:**
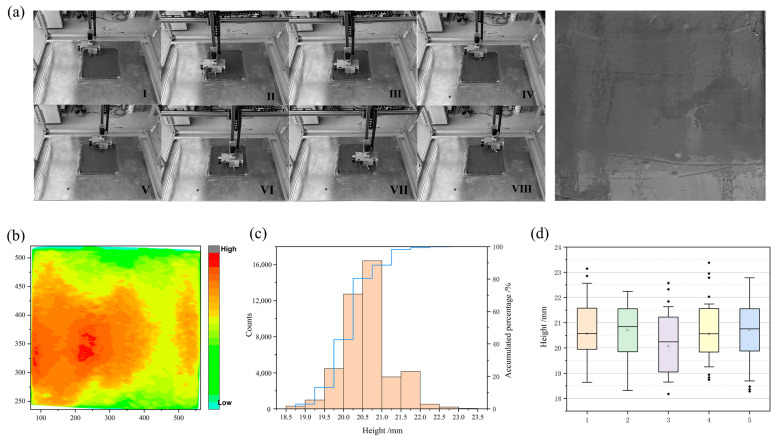
The results of the surface smoothing operation on concrete without embedded components: (**a**) real experimental results; (**b**) depth image of concrete surface height; (**c**) distribution of surface height; (**d**) distribution of surface height across five experimental groups.

Based on the relative position of the processing tooling to the robot’s origin, environmental mapping was conducted, integrating with the path map depicted in Figure 8 to obtain relevant information regarding robot processing. The actual trajectory of the robot during smoothing operation is depicted in Figure 12a, with I–VIII denoting key positions of the smoothing operation. Using the origin of the tooling and the positive direction of the X-axis joint as the starting point and initial forward direction of the end-effector, one cycle of smoothing operation ranged from 120 s to 125 s. It can be observed from the planned trajectory and the final concrete surface that the robot effectively achieved comprehensive coverage for smoothing concrete surfaces, albeit with some localized seepage and traces left by the smoothing tool. This phenomenon may be related to the process parameters of concrete under different temperature and humidity conditions.

In Figure 12b, a depth image of the concrete surface height from the first experimental group is displayed. Based on the size of the mold, the best smoothing quality is achieved when the height is 20 mm. In Figure 12c, the left side shows the distribution of point cloud quantities in different height ranges, while the right side shows the cumulative percentage of current height values over all data. It can be observed from the diagram that when the height data falls within the [18.5 mm, 19.5 mm] range, the cumulative percentage of the point cloud quantity does not exceed 10%. However, when the height data is in the [19.5 mm, 21.0 mm] range, the cumulative percentage of the point cloud quantity reaches 74.7%. Thus, the surface height values are primarily concentrated in the [19.5 mm, 21.0 mm] range. Through calculation, the overall height mean is 20.56 mm, with a standard deviation of 0.6. Figure 12d displays quantitative statistical data obtained from five repeated experiments (data for each experiment are shown in Table 4). The height data from each experiment is generally concentrated within the [19 mm, 21.5 mm] range. The overall deviation of the height mean from five experiments does not exceed 1 mm, and the standard deviation of each set of data is less than 1. This generally meets the allowable deviation range for smoothing operations of prefabricated components.

#### 4.2.2. With Embedded Parts

**Figure 13:**
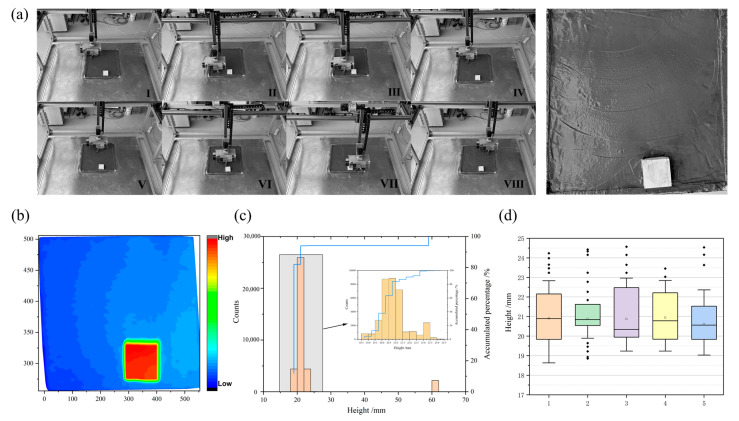
The results of the surface smoothing operation on concrete with embedded components: (**a**) real experimental results; (**b**) depth image of concrete surface height; (**c**) distribution of surface height; (**d**) distribution of surface height across five experimental groups.

Figure 13a shows the actual experimental results of smoothing with embedded components. It can be observed from Figure 13a that the robot can effectively avoid obstacles and complete the smoothing operation with embedded components, validating the feasibility of the algorithm. The depth image is depicted in Figure 13b. Statistical data of surface height are shown in Figure 13c, indicating that the heights of the embedded components mainly range from [59.5 mm, 62 mm]. When calculating surface smoothness, data from this part need to be excluded. After processing, the calculated height mean is 20.91 mm, with a standard deviation of 0.97. Compared to the smoothing effect without embedded components, the proportion within the range [22 mm, 24 mm] is 7.87%. Further analysis reveals that when the smoothing fan approaches the embedded component, there is a blind spot in the operation between the smoothing fan and the embedded component. The tool may accumulate a small amount of concrete residue in the blind spot, leading to a higher surface height in that area. To quantitatively analyze the smoothing effect without embedded components, we conducted visual processing on five experiments, as shown in Figure 13d, and the final results are presented in Table 5. The average of the height mean values from the five experiments is 20.84 mm.

### 4.3. Actual Testing of Roughening Experiments

**Figure 14:**
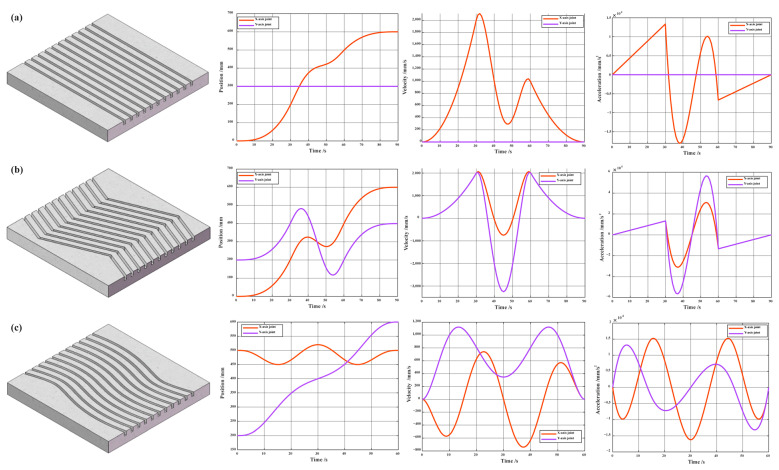
Variations in position, velocity, and acceleration of each joint under different texture processing: (**a**) linear type; (**b**) polyline type; (**c**) B-spline curve type.

Figure 14 depicts the trajectory plots of different roughening texture patterns, as well as the variations in joint position, velocity, and acceleration. Figure 14a,b utilize a 3-5-3 segmented trajectory planning, while Figure 14c employs B-spline trajectory planning. It is evident from the figures that regardless of the trajectory planning method used, the joint velocities exhibit smooth transitions, and there are no sudden changes in acceleration.

**Figure 15:**
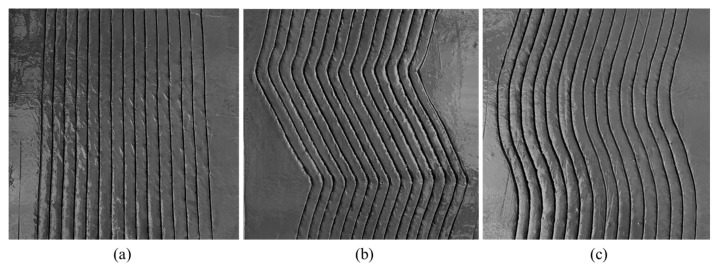
The final result image of roughening: (**a**) linear type; (**b**) polyline type; (**c**) B-spline curve type.

Based on the trajectory planning simulation results described above, roughening texture processing was conducted on the actual prototype. Figure 15 depicts the final experimental results of roughening under three different trajectories. The formation of this texture processing path and the process of texture formation intuitively demonstrate the feasibility of the designed configuration and trajectory planning. In Figure 15a, the final roughening texture surface with B-spline curve type exhibits slight flipping and cracking, which is related to the inclination angle of the trowel tool and the roughening speed. In Figure 15b, the polyline texture exhibits bending at sharp points, which is related to the pushing force of the trowel against the concrete contact surface and the strength of the trowel material. In the subsequent research process, the angle between the tool and the concrete surface during roughening can be adjusted in real-time based on the different concrete surface conditions. Additionally, an analysis of roughening effects can be conducted for different trowel materials. Furthermore, adding a vibration module can reduce the bonding effect between the concrete surface and the trowel, thereby achieving better roughening results.

## 5. Conclusions

This paper explores the smoothing and roughening processes in the production of precast concrete components and successfully develops a robot system integrated with an intelligent end-effector for these tasks. The system facilitates automated integration of both smoothing and roughening processes, significantly improving the production efficiency of precast components. The study also introduces a full-coverage smoothing path planning method based on a biologically inspired neural network and a diverse approach to robotic roughening path planning. Notably, the system can promptly adjust its path using the A* algorithm when encountering dead zones. The proposed full-coverage smoothing path planning algorithm is compared with the traditional A* algorithm. Experimental results on a 25 × 25 grid map show that while maintaining overall coverage, the new algorithm reduces the path length by 24 cm and lowers the overall repetition rate by 3.3%. However, there may still be room for improvement in optimizing the number of turns.

Additionally, to verify the feasibility and effectiveness of the proposed system and its operational methods, smoothing experiments were conducted with and without embedded components, along with roughening experiments tailored to straight, polyline, and curved texture requirements. Quantitative analysis of the smoothed surfaces using specialized measuring equipment revealed that the overall height deviation of the smoothed concrete surfaces was less than 1 mm, and the standard deviation was also less than 1. Furthermore, the three different textural forms of roughening achieved the expected results, further confirming the practicality and efficiency of the smoothing and roughening robot system.

Our future work will focus on the following aspects: (1) the impact of different material characteristics on smoothing and roughening effects: selecting suitable tool materials for different materials. Furthermore, the study can explore tool wear mechanisms and cutting forces to improve tool efficiency and reduce processing costs. (2) Expanding new functions of the robotic system. For example, intelligent handling of different process parameters, material ratios, and environmental conditions. (3) Application of automation technology: combining advanced technologies such as machine vision and machine learning to research how to achieve automated control and intelligent monitoring of the smoothing and roughening processes. For instance, developing an intelligent smoothing and roughening system capable of adaptive adjustments based on real-time collected data, improving processing accuracy and consistency.

## Figures and Tables

**Table 1:** The final error results.

Axis Number	Group 1	Group 2	Group 3	Group 4	Group 5
X-axis	0.15 mm	0.13 mm	0.12 mm	0.15 mm	0.15 mm
Y-axis	0.14 mm	0.16 mm	0.15 mm	0.14 mm	0.15 mm
Z-axis	0.14 mm	0.14 mm	0.16 mm	0.15 mm	0.17 mm

**Table 2:** The detection results.

Corner Number	Actual Position (mm)	Test Position (mm)	Position Error (mm)
1	(420, 205, 20)	(419.73, 205.46, 19.75)	0.98
2	(970, 205, 20)	(970.25, 205.21, 19.87)	0.59
3	(420, 605, 20)	(420.24, 604.86, 20.55)	0.93
4	(970, 605, 20)	(970.31, 604.77, 20.32)	0.86

**Table 3:** The final comparative results between the two algorithms.

Path Planning Methods	Coverage (%)	Path Length (cm)	Path Overlap Rate (%)
A* algorithm on a 15 × 15 grid map	99.56	227.2	8
BINN + A* algorithm on a 15 × 15 grid map	100	216.3	5.8
A* algorithm on a 25 × 25 grid map	99.21	605	5.6
BINN + A* algorithm on a 25 × 25 grid map	100	581	2.3

**Table 4:** Surface smoothness data for five sets of smoothing experiments without embedded components.

Group Number	Minimum (mm)	Maximum (mm)	Average (mm)	Standard Deviation
1	18.64	23.15	20.56	0.60
2	18.32	22.24	20.70	0.67
3	18.18	24.12	20.06	0.82
4	18.74	23.38	20.54	0.58
5	18.28	22.78	20.70	0.71

**Table 5:** Surface smoothness data for five sets of smoothing experiments with embedded components.

Group Number	Minimum (mm)	Maximum (mm)	Average (mm)	Standard Deviation
1	18.63	24.23	20.91	0.97
2	18.85	24.42	20.87	0.65
3	19.23	24.57	20.88	0.96
4	19.24	23.45	20.94	0.87
5	19.03	24.54	20.61	0.76

## Data Availability

Data are contained within the article.
